# Immediate Modulation of Transcutaneous Auricular Vagus Nerve Stimulation in Patients With Treatment-Resistant Depression: A Resting-State Functional Magnetic Resonance Imaging Study

**DOI:** 10.3389/fpsyt.2022.923783

**Published:** 2022-07-01

**Authors:** Jifei Sun, Yue Ma, Zhongming Du, Zhi Wang, Chunlei Guo, Yi Luo, Limei Chen, Deqiang Gao, Xiaojiao Li, Ke Xu, Yang Hong, Fengquan Xu, Xue Yu, Xue Xiao, Jiliang Fang, Xiaobing Hou

**Affiliations:** ^1^Guang’anmen Hospital, China Academy of Chinese Medical Sciences, Beijing, China; ^2^Graduate School of China Academy of Chinese Medical Sciences, Beijing, China; ^3^Dongzhimen Hospital, Beijing University of Chinese Medicine, Beijing, China; ^4^Beijing First Hospital of Integrated Chinese and Western Medicine, Beijing, China

**Keywords:** treatment-resistant depression, taVNS, rs-fMRI, amplitude of low-frequency fluctuations, functional connectivity

## Abstract

**Background:**

Previous studies found that transcutaneous auricular vagus nerve stimulation (taVNS) was clinically effective in treating a case of treatment-resistant depression (TRD). However, the brain neural mechanisms underlying the immediate effects of taVNS treatment for TRD have not been elucidated.

**Materials and Methods:**

Differences in the amplitude of low-frequency fluctuations (ALFF) between TRD and healthy control (HC) groups were observed. The TRD group was treated with taVNS for 30 min, and changes in ALFF in the TRD group before and after immediate treatment were observed. The ALFF brain regions altered by taVNS induction were used as regions of interest to analyze whole-brain functional connectivity (FC) changes in the TRD group.

**Results:**

A total of 44 TRD patients and 44 HCs completed the study and were included in the data analysis. Compared with the HC group, the TRD group had increased ALFF in the left orbital area of the middle frontal gyrus. After taVNS treatment, ALFF in the left orbital area of the middle frontal gyrus and right middle frontal gyrus decreased in the TRD group, while ALFF in the right orbital area of the superior frontal gyrus increased. The FC in the left orbital area of the middle frontal gyrus with left middle frontal gyrus and the right inferior occipital gyrus was significantly increased.

**Conclusion:**

Transcutaneous auricular vagus nerve stimulation demonstrates immediate modulation of functional activity in the emotional network, cognitive control network, and visual processing cortex, and may be a potential brain imaging biomarker for the treatment of TRD.

## Introduction

Major depressive disorder (MDD) is a common psychiatric disorder characterized by depressed mood, cognitive impairment, and somatic symptom disturbances (1). Epidemiological surveys show that approximately 350 million people worldwide are affected by MDD, and that by the end of 2030, MDD will become the number one disease burden worldwide (2, 3). Despite extensive studies, 30–40% of MDD patients do not respond significantly to treatment (4). The most typical definition of treatment-resistant depression (TRD) is that a patient does not improve after at least two or more treatments of adequate dose and duration (5). Compared with patients with non-TRD, patients with TRD incur higher medical costs, are twice as likely to be hospitalized, and are at a greater risk of suicide (6–8). Therefore, clinicians must address significant challenges in improving the clinical efficacy of TRD (9).

Currently, Several neuromodulation techniques, including repetitive transcranial magnetic stimulation (rTMS) (10), electroconvulsive therapy (11), deep brain stimulation (DBS) (12), and magnetic seizure therapy (13), are available to alleviate depressive symptoms and improve quality of life in patients with TRD. However, the presence of potential side effects, high costs, and complicated procedures limit their widespread application.

Vagus nerve stimulation (VNS) has received significant attention in clinical research as an antidepressant treatment technique. It was approved by the FDA in 2005 for the treatment of TRD and has relieved the suffering of many patients (14, 15). The implementation of VNS in the body stimulates the vagal afferent brainstem pathway associated with emotion regulation by sending electrical impulses to the left cervical vagus nerve, which consequently regulates brain areas associated with emotion regulation (16, 17). Although VNS is generally well tolerated and can improve the mood of a patient, the risk of anesthesia and surgery must be considered. An overall complication rate of up to 12% and surgical complication rate of up to 8.6% have been reported for VNS, which hinder its more extensive clinical use in TRD patients (18).

A non-invasive method of VNS, namely, transcutaneous auricular vagus nerve stimulation (taVNS), has been developed to overcome the shortcomings of regular VNS (19). The auricular region is the main distribution area of the auricular branch of the vagus nerve. The peripheral vagus nerve mainly transmits signals to the solitary bundle nucleus, and then directly or indirectly affects the neurological activity of the brain through other brainstem structures, such as the locus coeruleus and parabrachial nucleus (20). Previous studies have shown that taVNS produces similar effects as VNS in improving depressive symptoms by stimulating the auricular vagus nerve and offers the advantage of zero pain, non-invasiveness, and portability (21–23). TaVNS can also clinically significantly improve depression, anxiety, and drive symptoms in patients with TRD (24). Meanwhile, taVNS has been used for MDD and other disorders such as insomnia (25), disorders of consciousness (26), epilepsy (27), and migraine (28). However, the immediate brain effects of taVNS on TRD are yet to be elucidated.

In recent years, resting-state functional magnetic resonance imaging (MRI) (rs-fMRI) has been gradually applied in the fields of MDD (23), schizophrenia (29), autism (30), and other psychiatric disorders, and the amplitude of low-frequency fluctuation (ALFF) and functional connectivity (FC) have been investigated using rs-fMRI. ALFF reflects the intensity of spontaneous activity in a region of the brain over a short duration based on the calculated average amplitude of low-frequency oscillations in that region (31). FC is investigated based on interactions within and between brain networks *via* the analysis of correlations using time series between seed points or regions of interest (ROI) and whole brain voxels (32). The combination of ALFF and FC enable the disease to be investigated more comprehensively.

Previous studies have shown that TRD is associated with abnormal FC in the cognitive control network (CCN), default mode network (DMN), and salience network (SN) (33, 34). Previously, we showed that taVNS can alleviate the clinical symptoms of patients with MDD by modulating the function of the DMN and SN (23, 35, 36). A recent case report study showed that taVNS significantly improved depressive symptoms in patients with TRD after 8 weeks of treatment, and the mechanism of efficacy may be related to the modulation of FC between the rostral anterior cingulate cortex (rACC) and precuneus, insula, and dorsolateral prefrontal cortex (DLPFC) by taVNS (37). However, the mechanism underlying the immediate brain effect of taVNS on TRD is yet to be elucidated.

Therefore, in this study, the immediate pre- and post-ALFF and FC changes in patients with TRD treated with taVNS are observed using rs-fMRI. We hypothesize that taVNS may immediately modulate spontaneous neuronal activity in specific brain regions of TRD patients, which will provide insights into clinical studies of taVNS for TRD.

## Materials and Methods

### Subjects

The TRD patients for this study were recruited from Guang’anmen Hospital of the Chinese Academy of Chinese Medicine Sciences, Beijing First Hospital of Integrated Chinese and Western Medicine, and Xuanwu Hospital of the Capital Medical University. All patients with TRD showed the initial diagnosis of MDD in the fifth edition of the American Diagnostic and Statistical Manual of Mental Disorders (DSM-V, 2015). The inclusion criteria were as follows: (1) age, 18–70 years; (2) 17-item Hamilton depression rating scale (HAMD-17) score > 17, (3) right-handedness, and (4) no response to two or more adequate doses and courses of antidepressant therapy. Fifty gender- and age-matched healthy controls (HCs) (32 women and 18 men) were included in the HC group, which reflected the following: (1) age, 18–70 years; (2) HAMD-17 score < 7; (3) right-handedness; (4) no history of any mental illness in first-degree relatives.

The exclusion criteria for the patients and HCs were as follows: (1) serious mental illness and other diseases such as cardiovascular and cerebrovascular disorders; (2) history of drug and alcohol abuse; (3) contraindications to MRI, such as the presence of a heart pacemaker, metal fixed false teeth, or severe claustrophobia; (4) pregnancy or lactating; and (5) bipolar disorder or suicidal ideation; (6) previous participation in electrical stimulation therapy.

### Study Design

**FIGURE 1:**
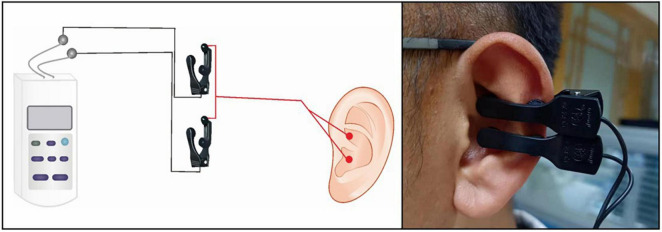
The study diagram shows the site of taVNS on the auricular surface.

**FIGURE 2:**
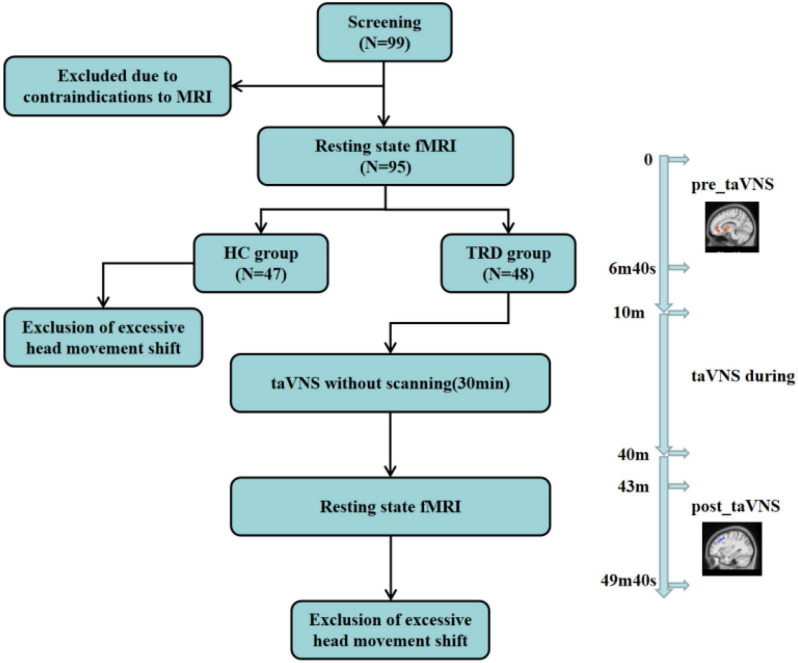
This figure reflects to the experimental design, the subjects’ fMRI scans, the TRD patients treated with taVNS, and the exclusions. Resting-state brain imaging data were acquired using 3.0 T magnetic resonance imaging (MRI) at 3 min and 20 s before (“pre_taVNS”) and 3 min after (“post_taVNS”) taVNS. The taVNS was treated for 30 min immediately in the middle of the fMRI scan.

A taVNS device (Hwato, SDZ-IIB, Suzhou, China) was used to stimulate the patients, and the stimulation site was the patient’s bilateral auricular concha area. Based on previous studies, the following parameters were used in the experiments (35–37): (1) frequency of 20 Hz, with wave width of less than 1 ms, and (2) intensity of 4–6 mA (Adjusted to patient tolerance, relatively moderate). The duration of the stimulation was 30 min (Figures 1, 2).

### Image Acquisition

All patients were subjected to MRI using a Magnetom Skyra 3.0-T scanner (Siemens, Erlangen, Germany). Before scanning was performed, the patients were instructed to remain awake and avoid active thinking. During scanning, the patients were required to wear earplugs and noise-canceling headphones, use a hood to immobilize their head, and lie flat on the examination bed. The scanning procedure involved a localizer scan, high-resolution three-dimensional T1-weighted imaging, and BOLD fMRI.

The scanning parameters were as follows: for three-dimensional T1-weighted imaging, the time repetition/time echo = 2500/2.98 ms, flip angle = 7°, matrix = 64 × 64, field of view = 256 mm × 256 mm, slice thickness = 1 mm, slice number = 48, slices = 192, and scanning time = 6 min, 3 s; for BOLD-fMRI, the time repetition/time echo = 2000/30 ms, flip angle = 90°, matrix = 64 × 64, field of view = 240 mm × 240 mm, slice number = 43, slice thickness/spacing = 3.0/1.0 mm, number of obtained volumes = 200, and scanning time = 6 min, 40 s (Figure 2).

### Image Processing

#### Functional Magnetic Resonance Imaging Data Preprocessing

The acquired rs-fMRI data were preprocessed using MATLAB-based DPARSF 5.1 software (DPARSF 5.1^1^) (38), as follows: (1) conversion of DICOM raw data to NIFTI format; (2) removal of the first 10 time points to stabilize the data; (3) slice timing; (4) realignment of head motion (removal of patients with head movements greater than 2 mm in any direction and motor rotation greater than 2°); (5) regression of covariates, including brain white matter signal, cerebrospinal fluid signal, and head movement parameters; (6) spatial normalization: functional images of all subjects were converted to the Montreal Neurological Institute (MNI) standard space using DARTEL; (7) resampling the functional images to 3 mm × 3 mm × 3 mm cubic voxels and smoothing functional images with a 6-mm Gaussian kernel of full width at half maximum.

### Amplitude of Low-Frequency Fluctuations Analysis

The data were spatially normalized and smoothed, and a fast Fourier transform was performed to switch the time series to the frequency domain to obtain the power spectrum. The square root of the power spectrum at each frequency was calculated to obtain the average square root of the ALFF measurement for each voxel within 0.01–0.08 Hz. Finally, bandpass filtering (0.01–0.08 Hz) was performed. To reduce inter-individual variability, the ALFF was transformed to zALFF using Fisher’s *Z-*transformation prior to statistical analysis.

### Functional Connectivity Analysis

To investigate changes in brain circuit connectivity in patients with taVNS-induced TRD, after smoothing and filtering the pre-processed data, the AAL template of WFU_Pick Atlas_v3.0 software was used for ROI extraction (39). Subsequently, the time series of the left orbital area of the middle frontal gyrus was extracted, and the correlation coefficient of the time series corresponding to the left orbital area of the middle frontal gyrus, and all voxels of the whole brain was calculated. Then the data was transformed with Fisher’s *z* to make it in normal distribution.

### Statistical Analysis

#### Clinical Data Analysis

Clinical data were analyzed using SPSS 23.0 statistical software (IBM Corporation, Somers, NY, United States). A two-sample *t*-test was used to compare the age, education level, and HAMD-17 scores between the two groups (the normality of the data was evaluated *via* the Shapiro–Wilk test), and the chi-square test was used to compare the gender between the two groups. The threshold for statistical significance was set at *P* < 0.05 (two tailed).

##### Functional Magnetic Resonance Imaging Data Analysis

Image data statistics were analyzed using DPARSF 5.0 software. The participants’ gender, age, education level, and framewise displacement (FD) metric (derived from Jenkinson’s formula) were used as covariates, and the ALFF of the TRD and HC groups were compared *via* a two-sample *t*-test. The participants’ FD metric was used as a covariate, and the paired-samples *t*-test was performed to compare the ALFF and FC of the TRD group before and after treatment. The ALFF and FC results were corrected using the Gaussian random field (GRF), and the corrected cluster levels were set at *P* < 0.05 (two tailed), whereas threshold voxel levels of *P* < 0.005 were defined as statistically different. Meanwhile, clusters comprising less than 60 voxels were removed.

## Results

### Characteristics of Research Samples

Ninety-nine participants were screened. Four participants quit early owing to claustrophobia or mental issues. Four TRD patients and one HC were excluded because of excessive head movements in the fMRI scan. Finally, 90 participants were considered (44 in the TRD group and 46 in the HC group) (Figure 2).

No statistical differences were observed between the two groups in terms of gender, age, and years of education. By contrast, a statistical difference was observed between the TRD and HC groups in terms of the HAMD-17 scores. The duration of disease in the TRD group was 42.36 ± 19.14 months (Table 1).

**TABLE 1:** Demographic and clinical variables.

Variable	TRD group (*n* = 44)	HC group (*n* = 46)	*t/χ* ^2^	*P*-value
Gender (M/F)	19/25	18/28	0.152	0.696^a^
Age (years)	44.13 ± 12.02	41.89 ± 12.91	0.853	0.396^b^
Education (years)	13.90 ± 3.40	13.36 ± 3.43	0.748	0.457^b^
Duration of illness (months)	42.36 ± 19.14	NA	NA	NA
HAMD-17 score	23.09 ± 3.12	1.06 ± 0.80	46.270	<0.001^b^*

*TRD, treatment resistant depression; HC, healthy control; HAMD-17, 17-item Hamilton Rating Scale for Depression; NA, not applicable.*

*^a^The P-values of gender distribution between the two groups were obtained by chi-square test.*

*^b^The P-value obtained by two-sample t-test.*

*Significant difference.

### Functional Magnetic Resonance Imaging Results

#### Differences in Amplitude of Low-Frequency Fluctuations Between Treatment-Resistant Depression and Healthy Control Groups

**FIGURE 3:**
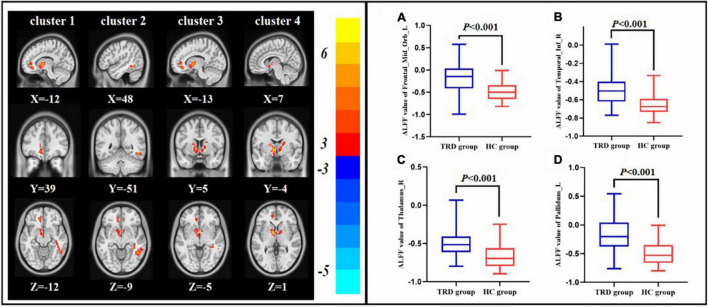
Difference in ALFF between the pre-treatment TRD and HC groups. ALFF value of left orbital area of the middle frontal gyrus **(A)**, right inferior temporal gyrus **(B)**, right thalamus **(C)**, and right angular **(D)**.

Compared with the HC group, the TRD group indicated an elevated ALFF in the left orbital area of the middle frontal gyrus, right inferior temporal gyrus, right thalamus, and left pallidum (GRF correction, Table 2 and Figure 3).

**TABLE 2:** Differences in ALFF between the TRD and HC groups; changes in ALFF and FC after taVNS treatment in the TRD group.

Clusters	Brain regions	MNI peak			Voxel size	*t*-value (peak)
		X	Y	Z		
**TRD group vs. HC group**						
1	Left orbital area of the middle frontal gyrus	–12	39	–12	65	4.629
2	Right inferior temporal gyrus	48	–51	–9	89	5.038
3	Right thalamus	7	–4	1	118	3.134
4	Left pallidum	–13	5	–5	170	3.694
**Post_ALFF vs. Pre_ALFF**						
1	Left orbital area of the middle frontal gyrus	–24	39	–9	116	–2.965
2	Right orbital area of the superior frontal gyrus	15	36	–18	82	4.255
3	Right middle frontal gyrus	33	12	51	130	–2.963
**Post_FC vs. Pre_FC**						
1	Left middle frontal gyrus	–33	57	12	90	3.954
2	Right inferior occipital gyrus	36	–81	–12	138	3.307

*Pre, before taVNS treatment; Post, after taVNS treatment; ALFF, amplitude of low-frequency fluctuation; FC, functional connectivity; MNI peak, coordinates of primary peak locations in the Montreal Neurological Institute space.*

### Comparison of Post-treatment and Pre-treatment Amplitude of Low-Frequency Fluctuations in Treatment-Resistant Depression Group

**FIGURE 4:**
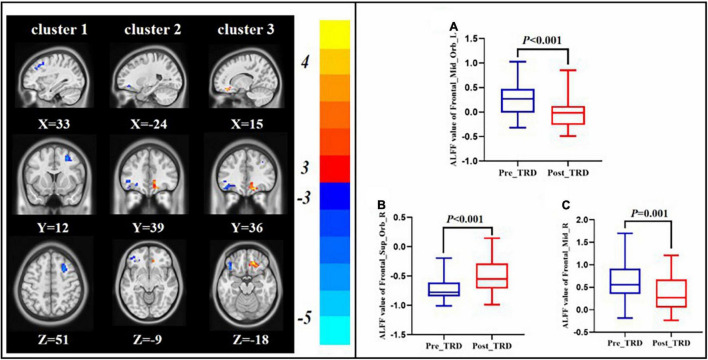
Changes in ALFF between the post-treatment and pre-treatment. ALFF value of left orbital area of the middle frontal gyrus **(A)**, right orbital area of the superior frontal gyrus **(B)**, right middle frontal gyrus **(C)**.

Compared with the pre-treatment TRD group, the post-treatment TRD group indicated increased ALFF in the right orbital area of the superior frontal gyrus and decreased ALFF in the right middle frontal gyrus and left orbital area of the middle frontal gyrus (GRF correction, Table 2 and Figure 4).

### Comparison of Post-treatment and Pre-treatment Functional Connectivity in the Treatment-Resistant Depression Group

**FIGURE 5:**
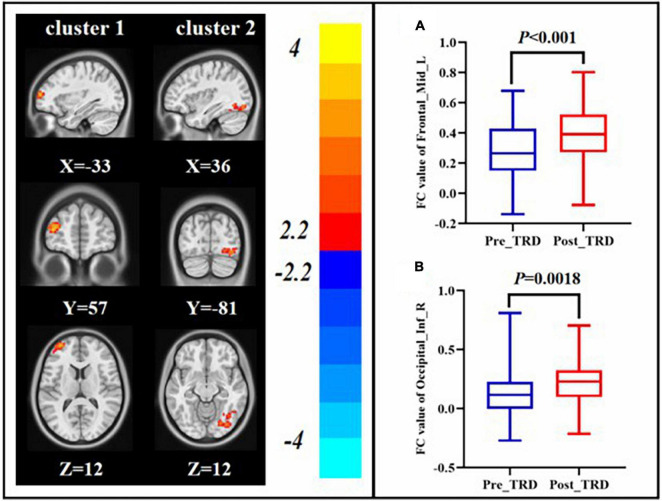
Changes in FC between the post-treatment and pre-treatment. FC value of left middle frontal gyrus **(A**), right inferior occipital gyrus **(B)**.

Interestingly, we found that the difference in ALFF between the TRD group immediately before and after treatment with between the TRD and HC group partially overlapped in the left orbital area of middle frontal gyrus. Therefore, we chose the left orbital area of middle frontal gyrus as the ROI to observe changes in brain circuit FC in patients with TRD treated with taVNS. After taVNS treatment, the FC of left orbital part of middle frontal gyrus with left middle frontal gyrus and right inferior temporal gyrus was significantly elevated in TRD group (GRF correction, Table 2 and Figure 5).

## Discussion

To our knowledge, this is the first study using rs-fMRI to observe the mechanism of the immediate effect of taVNS treatment in patients with TRD. The results of this study showed that the cortico-striatal-pallidal-thalamic (CSPT) circuit was abnormal in the TRD group compared to the HC group. After 30 min of immediate treatment with taVNS in the TRD group, ALFF was reduced in the left orbital area of the middle frontal gyrus and right middle frontal gyrus, but increased in the right orbital area of the superior frontal gyrus. In addition, the ROI-based FC between the left orbital area of the middle frontal gyrus with left middle frontal gyrus and right inferior temporal gyrus was significantly increased.

We discovered that the TRD group indicated increased ALFF in the left orbital area of the middle frontal gyrus, right inferior temporal gyrus, right thalamus, and left pallidum as compared with the HC group. In recent years, researchers discovered that patients with depression exhibit a wide distribution of localized abnormal brain functional areas as compared with HCs, and these abnormal brain areas are located in the previously proposed neuropathological circuit of depression, namely, the CSPT neurotransmission pathway (40–42). The above mentioned circuit is composed of distributed neuroanatomical loops that connect the prefrontal cortex, basal ganglia, and thalamus in an integrated manner to support different emotional, cognitive, and motor processes (43, 44). The results of this study suggest that most of these brain regions are involved in the CSPT circuit. Previous studies have shown that the CSPT loop is an important target for DBS in the treatment of TRD (12, 45). VNS modulates brain regions that are directly related to emotion, including the amygdala, thalamus, and cerebral cortex (which constitute the CSPT circuit), through the solitary nucleus, which subsequently necessitates the treatment of TRD. These findings provide a mechanistic basis for the taVNS treatment of TRD (46).

In this study, we discovered that the TRD group exhibited higher ALFF in the left orbital area of the middle frontal gyrus as compared with the HC group. Interestingly, the ALFF in the left orbital area of the middle frontal gyrus decreased in the TRD group immediately after the treatment. The left orbital area of the middle frontal gyrus is located in the prefrontal lobe and constitutes the reward network (47, 48). A core symptom of TRD is the inability to feel pleasure, and abnormalities in the reward network are important in the pathogenesis of TRD (49). The orbital frontal cortex (OFC) participates in emotion regulation, sensory integration, pain regulation, and reward prediction in the human body (50, 51). Additionally, previous studies indicate that patients with TRD exhibit higher ALFF in the frontal lobe than HCs (52). Therefore, the results of this study suggest that the abnormal activation of the OFC in TRD may be an important pathological mechanism and that the DBS stimulation of the nucleus accumbens in patients with TRD decreases metabolism in the subgenual cingulate and OFC (53). Another task-state case report regarding VNS for TRD shows a direct modulatory effect of VNS on brain regions such as the OFC and polar prefrontal cortices in patients with TRD (54). Therefore, the results of the present study suggest that taVNS exerts an immediate modulatory effect on the OFC in TRD patients and further suggests that taVNS downregulates the emotion and reward network, which may be a key mechanism by which taVNS functions in patients with TRD.

After taVNS treatment was performed, we discovered that the ALFF in the right middle frontal gyrus decreased in the TRD group, whereas the FC of the left orbital area of the middle frontal and left middle frontal gyrus increased. The middle frontal gyrus is located in the DLPFC and is an important component of the CCN. The CCN is vital to human emotion regulation, cognitive activity, and behavioral functions (55, 56). Meanwhile, the DLPFC determines the negative emotion and behavior control (57, 58). Previous studies have shown reduced FC between the DLPFC and angular gyrus in patients with TRD, suggesting that reduced FC between the CCN and DMN is a mechanism in the pathogenesis of TRD (33). In our previous study, we discovered that symptom improvement in patients with TRD was associated with an increase in the FC of taVNS-induced rACC and DLPFC (37). Therefore, we speculated that the immediate modulatory effect of taVNS on the CCN of TRD may constitute its action mechanism.

Furthermore, we discovered that the FC of the right inferior occipital gyrus increased in the TRD group after immediate treatment with taVNS. The right inferior occipital gyrus belongs to the visual cortex, which primarily acquires and processes visual information, as well as participates in mental activities such as emotion and attention (59, 60). Abnormalities in the functional activity of neurons in the visual cortex of patients with TRD has been reported previously (61). Previously, we discovered that the FC of the right rACC and occipital lobe increased in patients with TRD after 2 months of treatment as compared with during the pre-treatment period (37). Meanwhile, in two different studies, immediate and long-term modulatory effects of taVNS on the function of vision-related cortex in patients with primary insomnia were demonstrated (25, 62). Therefore, we speculate that the immediate modulation of the visual cortical function by taVNS in patients with TRD may contribute to its neuromechanism.

## Limitation

This study presents a few limitations. First, it is preferable to treat TRD with taVNS in the absence of antidepressants; however, for ethical reasons, the patients did not discontinue their use of antidepressants before enrollment. Therefore, the results of this study may be affected by potential pharmacological factors. Second, a sham taVNS group was not established for randomized control in this study, which further demonstrates that the change in outcome in the TRD group was due to taVNS. Third, This study only compared the differences between TRD and HC, but this study did not include the nTRD group. Therefore, the results for brain regions with increased ALFF (TRD vs. HC) only suggest that they are related to MDD but not to the pathophysiology of TRD, and further studies are needed in the future. Finally, the treatment parameters and immediate treatment time settings were based on previous experiments with MDD and TRD. Meanwhile, there is no uniformity in the selection of daytime morning or afternoon immediate treatment. Therefore, the settings of treatment parameters and treatment duration need to be further optimized and harmonized in the future.

## Conclusion

The present study used rs-fMRI technique to initially find that the pathogenesis of TRD is associated with abnormal CSPT circuits. taVNS has a direct modulatory effect on the functional activity of the emotional and reward network, CCN and parts of the visual processing cortex, indirectly improving abnormal CSPT circuits in TRD patients, which may be a potential brain focus for its treatment of TRD. This may provide some scientific data and references for the long-term treatment of patients with TRD by taVNS.

## Data Availability Statement

The raw data supporting the conclusions of this article will be made available by the authors, without undue reservation.

## Ethics Statement

The experimental protocol was approved by the Ethics Committee of Guang’anmen Hospital, China Academy of Chinese Medical Science (No. 2017-021-SQ), Trial registration. China Clinical Trials Registry. chiCTR1800014277. All patients signed an informed consent form before enrollment. The patients/participants provided their written informed consent to participate in this study. Written informed consent was obtained from the individual(s) for the publication of any potentially identifiable images or data included in this article.

## Author Contributions

JF conceived and designed the experiments and revised the manuscript. JS collected cases, analyzed data, and wrote the manuscript. YM, ZD, ZW, CG, YL, LC, DG, XL, and KX collected cases and analyzed data. YH scanned the subjects. XH, XY, and XX evaluated patients and collected cases. All authors contributed to the article and approved the submitted version.

## Conflict of Interest

The authors declare that the research was conducted in the absence of any commercial or financial relationships that could be construed as a potential conflict of interest.

## Publisher’s Note

All claims expressed in this article are solely those of the authors and do not necessarily represent those of their affiliated organizations, or those of the publisher, the editors and the reviewers. Any product that may be evaluated in this article, or claim that may be made by its manufacturer, is not guaranteed or endorsed by the publisher.

## Footnotes

1
http://www.rfmri.org/DPARSF
